# Improving hen welfare on cage-free egg farms in Asia: Egg producers’ perspectives

**DOI:** 10.1017/awf.2023.85

**Published:** 2023-09-22

**Authors:** Kate Hartcher, Jayasimha Nuggehalli, Qing Yang, Maria Catalina T. de Luna, Ali Agus, Shuichi Ito, Zulkifli Idrus, Iman H.S. Rahayu, Jutamart Jattuchai, Kris Descovich, Elissa Lane, Michelle Sinclair

**Affiliations:** 1 School of Veterinary Science, University of Queensland, Brisbane, QLD, Australia; 2 Global Food Partners, Singapore; 3 Animal Law & Policy Program, Harvard Law School, USA; 4 Humane and Sustainable Food Lab, School of Medicine, Stanford University, Palo Alto, CA, United States; 5 Royal (Dick) School of Veterinary Studies, The University of Edinburgh, UK; 6 University of the Philippines Los Baños, Philippines; 7 Gadjah Mada University, Indonesia; 8 Tokai University, Japan; 9 Institute of Tropical Agriculture and Food Security, Universiti Putra Malaysia, Malaysia; 10 Bogor Agricultural University, Indonesia; 11 Chulalongkorn University, Thailand

**Keywords:** animal welfare, cage-free, egg producers, egg production, layer hens, poultry

## Abstract

There is a trend towards the adoption of cage-free housing systems in the egg industry across Asia. While cage-free housing systems can hold significant animal welfare advantages over cages, there can also be challenges in managing these systems. This exploratory study aimed to investigate the perspectives of egg producers on the main challenges and proposed solutions associated with cage-free systems in China, Indonesia, Thailand, Japan, Malaysia, and the Philippines. Cage-free producers found disease prevention and maintaining a healthy profit margin more difficult than producers from cage farms, while it was less difficult to provide environmental enrichment in cage-free systems compared to cage farms. The top challenges for cage-free producers were the cost of production, system management, disease, sales, and egg production, and the top proposed solution was to improve on-farm practices and efficiencies. Eighty-one percent of egg producers believed that more support is needed to maintain their farms than is currently available, and support was most needed in helping to improve sales, improve farm operations, lower farm costs, and provide information for producers in the form of education and training. Most responses identified the government as the stakeholder that should offer support. These results may help direct further studies in this field as well as supplying information to develop relevant initiatives with an emphasis on education and training, thereby improving animal welfare on cage-free farms and increasing the uptake of high welfare cage-free farms across the region.

## Introduction

Asia Pacific countries produce approximately two-thirds of the global egg supply (PoultryWorld 2021), with China alone producing over 40% of the world’s eggs (Yang 2021). Asia has accounted for over 70% of global growth in egg production, and this trend is expected to continue with developing economies in the region (WattPoultry 2021). The vast majority of hens in Asia are kept in conventional cages without furnishings, as opposed to the furnished cages used elsewhere in the world or cage-free systems. For example, a conservative estimate for China is that 90% of hens are housed in conventional cages (Yang 2020). Scientific findings combined with increasing public awareness of farm animal welfare has led many large companies to make global commitments to source only cage-free eggs, and the European Union is considering banning all cages (European Commission 2021). The potential for cage-free egg production is less documented in developing countries (Rodenburg *et al.* 2022), but a trend towards the adoption of cage-free egg housing systems is emerging across Asia (Compassion in Food Business 2021). That said, despite many cage-free commitments, only 44% of the 18 companies with Asia-Pacific cage-free commitments had reported progress as of 2021 (Compassion in Food Business 2021), and of the publicly reported sourcing on Welfare Progress, there is still a low rate of cage-free egg procurement (Welfare Progress 2023).

While there are advantages of cage systems, including lower mortality rates and less variable flock health, these are also seen in furnished cages which also provide behavioural opportunities (Hartcher & Jones 2017; Hemsworth 2021). It has been well-documented that conventional cages thwart highly motivated behaviours and reduce hens’ quality of life (Rodenburg *et al.* 2022). Indeed, the Welfare Footprint Project (2022) aimed to quantify the welfare impact of conventional and furnished cages compared to indoor aviary systems and found that in terms of time spent in pain, cage-free aviaries were superior to cage systems.

Despite the potential welfare advantages, the management of cage-free systems is generally more challenging than in cage systems which has contributed to a slower uptake than may otherwise have occurred (Rodenburg *et al.* 2022). Challenges in cage-free housing systems can include preventing and managing infectious diseases and parasites, the greater incidence of skeletal injuries incurred throughout the birds’ lives, and the prevention and control of injurious pecking (Groves 2021; Hemsworth 2021; Shifaw *et al.* 2023). In countries that have been adopting cage-free systems in recent decades, namely those in Europe and North America, there is evidence that issues can be reduced with increasing experience in managing the systems, and that mortality in cage and cage-free systems can reach comparable rates (Schuck-Paim et al. 2021).

It is important for countries that are yet to extensively adopt cage-free systems to identify the issues in Europe and improve the systems for domestic adoption (Singh & Groves 2021). For example, two of the biggest health and welfare problems in cage-free systems are the incidence of fractures incurred during the laying period, and injurious pecking. These problems may be mitigated by design, placement and management aspects of perches and housing, and flock management and genetic selection, respectively (Hartcher & Jones 2017; Singh & Groves 2021).

There is a need to focus on solutions to the known challenges in cage-free systems and involve the relevant stakeholders, including researchers, egg producers, and international animal protection non-government organisations (Rodenburg *et al.* 2022). It has been suggested that more value should be attached to practice-led innovations and advisor-focused initiatives to improve animal welfare and implement best practices (Van Dijk *et al.* 2019). Sinclair and Phillips (2019) stated that, historically, in certain areas of the world, important stakeholders are seldom consulted regarding animal welfare in livestock industries. Through a series of focus groups, interviews and surveys, they demonstrated that engaging with livestock industries can uncover mutual benefits for various stakeholders and solutions to challenging animal welfare issues (Sinclair & Phillips 2019).

The present study aimed to investigate the perspectives of egg producers on the main challenges and proposed solutions associated with cage-free systems in China, Japan, Indonesia, Malaysia, Philippines, and Thailand. Questions were also asked regarding the principal reasons for adopting cage-free systems.

## Materials and methods

### Ethical approval

This research was granted ethical approval through the University of Queensland, Australia (#2020002225).

### Data collection

This was conducted between January and June 2021. Country collaborators were involved to localise the study, invite participants from the local industries, facilitate translation, and to contribute to publications. No monetary compensation was provided for their participation. Methods for this study included: (1) Identifying academics in the respective focus countries and sending formal invitations to invite them to participate as in-country collaborators; (2) preparing questionnaires, an electronic platform, selection criteria for participants, and translator services; (3) distributing questionnaires to egg producers in each country via specific country collaborator(s); (4) collecting data; and (5) translating and analysing responses.

### Participants

Countries included in the study were China, Indonesia, Japan, Malaysia, the Philippines, and Thailand. These countries were chosen due to the size of their egg industries, as well as the level of support available – these countries have large egg industries and despite engaging in the early stages of adopting cage-free systems, little support is available. Each country collaborator aimed to elicit responses from at least ten cage and ten cage-free producers. Egg producers were eligible to participate in this study if they met the criteria shown in Table 1. Conventional cages were specified because furnished cages are not used in the region. There was a minimum size requirement of 10,000 hens for cage-free farms and not cage farms because the cage farm sizes are typically much larger than this.

**Table 1.** Egg producers were eligible to participate in this study if they met the participation criteria in this table

	Criterion	Requirement
Cage-free producers	Farm size	Minimum 10,000 hens
	Farming system	Any cage-free system. If farms have both cage and cage-free operations, they will be interviewed as cage-free
	Role	Engaged in a role that requires a technical awareness of on-farm operations, including the challenges and benefits of operating within the cage-free egg production system
	Length of service	Must have been working within the industry for a minimum of one year
**Cage producers**	Farm size	Representative of the size of cage farms in each country (all larger than 10,000 hens)
	Farming system	Conventional cages
	Role	Engaged in a role that has sufficient power within the organisation to make or contribute to decisions on transitioning to cage-free and knowledge of the operation
	Length of service	Must have been working within the industry for a minimum of one year

### Questionnaires

Collaborators contacted egg producers whom they were already in contact with, or had obtained the contact details for from local databases or industry associations. Producers were contacted via email with the study information, a consent form and a formal invitation to participate. Once the consent form had been received, an online questionnaire was sent in the producers’ local language, i.e. Chinese, Bahasa Indonesia, Japanese, English, Thai or Japanese. Responses were translated from the local languages to English for data analyses, and the questions were back-translated to check for accuracy. A mixed methodology approach was used; qualitative and quantitative questions were posed, with a primary emphasis on qualitative questions to investigate human attitudes. The questions relevant to this paper can be seen in the Supplementary material and are reported in the Results section. All questions were open-ended, apart from question 3, where producers were asked to rate the difficulty of certain aspects of egg production, and question 5 where producers were asked if more support is needed (yes, no, or maybe).

### Analysis

Data were anonymised by country collaborators before sending to three observers who were blind to all identifying information. These observers conducted the analysis. The data were compiled, coded, and cleansed. Binary and numerical data were summarised, and qualitative data were subjected to manual thematic analysis using software packages NVivo (QSR International 2018) and Microsoft Office®, where themes and subthemes were coded and described. If responses were unclear or not related to the question, they were omitted. Data within each theme were then subjected to further analysis to create subthemes according to their perceived intent, organised and quantified to understand the frequency and therefore the importance of theme and subtheme.

Statistical analyses of ordinal data (on a five-point scale) were carried out using ordinal logistic regression models (polr function; Venables & Ripley 2002) with R (R Core Team 2023) and RStudio (Posit team 2023) software. Housing system and country were fitted as nominal explanatory variables and model fitting was checked against null and full models using AIC and likelihood ratio tests (lrtest function; Zeileis & Hothorn 2002). Brants tests were used to check the proportional odds assumption (brant function; Schlegel & Steenbergen 2020) and fitting was also assessed using pseudo R^2^ values (Cox-Snell method) (PseudoR function; Signorell *et al.* 2023). Due to model assumptions and fitting, final models for five of the sixteen questions included housing system as the only explanatory variable.

## Results

A total of 224 egg producers were successfully recruited, and 202 (165 cage, 37 cage-free producers) participated through to the completion of the questionnaires. The number of cage-free producers that participated from each country were: Indonesia (n = 5); China (n = 9); Philippines (n = 10); Thailand (n = 8); Japan (n = 4); and Malaysia (n = 1). The response rate was 100% for every country except Thailand (60%). Since cage-free farms are scarce, country collaborators believed that the participants were highly representative of cage-free producers in each country. Cage-free producers that participated in this study (n = 37) either began with a cage-free system (n = 17), expanded a cage-based operation to include cage-free systems (n = 13), or transitioned entirely from a cage-based system to a cage-free one (n = 7). Participants operated a mix of free-range, barn and multi-tier aviary systems. The number of cage participants for each country were Indonesia (n = 103), China (n = 22), Philippines (n = 10), Thailand (n = 12), Japan (n = 10) and Malaysia (n = 8) with a total of 165 cage producers. Reflecting the current situation there are far fewer cage-free operations than cage farms in the focus countries and indeed the region, and the number of respondents in this study mirrored this, except the Philippines which had equal numbers of cage and cage-free participants.

**Figure 1.**
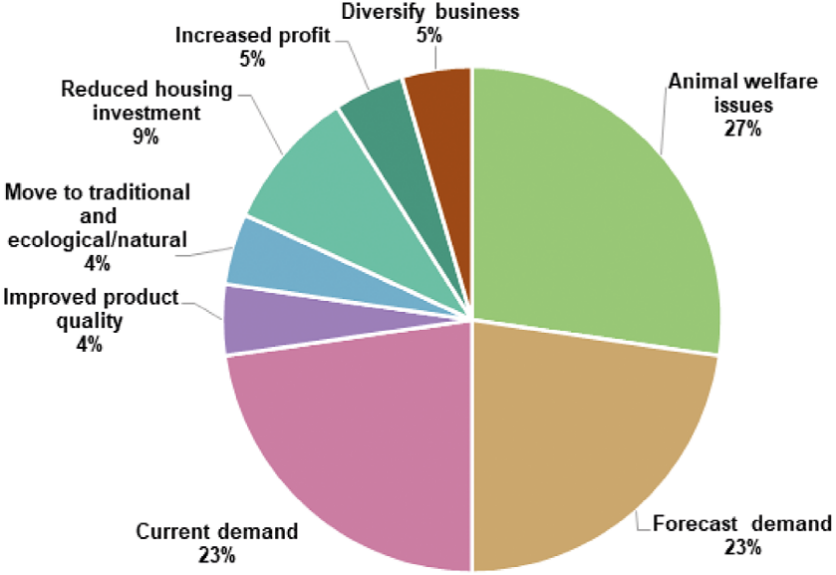
Qualitative responses by cage-free producers to the question: ‘Some cage egg farmers are changing to cage-free systems. What do you think are the reasons to use cage-free rather than cage systems?’ Summarised by thematic analysis across all countries.

We asked the cage-free producers an open-ended question to investigate why egg farmers are changing to cage-free systems, and the top responses were animal welfare and market demand (Figure 1).

### Challenges in maintaining cage and cage-free systems

Cage-free producers reported the main operational challenges to be the cost of production (22% of responses), followed by disease (14%), management of the system (14%), egg sales (13%), and rate of egg production (11%) (Table 2). Results are shown as the number of times that each topic came up in the producers’ responses, shown as the percentages of total answers. The cost of production comprised almost 60% of the answers in the Philippines. The Philippines also comprised half of the responses for system management. Thailand nominated the rate of egg production (mostly in the form of feed conversion ratio) as the top challenge, with most of the responses on this topic. In China, ‘product sales’ was the top challenge, while in Indonesia, it was disease and the cost of land.

**Table 2.** Cage-free egg producers’ answers to the open-ended question ‘What are the main operational challenges in running your cage-free farm?’ summarised by country

Emerging themes	Philippines	China	Indonesia	Japan	Malaysia	Thailand	Total
**Marketing/Market** Marketing/brand, distribution	3	2					5
**Product sales** Egg pricing, competitiveness of cage eggs	1	4		1		1	7
**Cost of production** Feed, operations, economies of scale, equipment	7		1		3	1	12
**Cost of land**			2		1		3
**Resourcing** Supply of litter and chicks from aviaries, staff challenges	1		1	1			3
**Disease** Management, biosecurity	1	2	2	1		2	8
**Production rate** Feed conversion ratio, egg production rate				1		5	6
**Floor eggs** Collection difficulties, hygiene	1	2		1			4
**Management of the system** Environment control, weather, husbandry, achieving growth rates, record keeping, feed calculation, knowledge of the system	4	2		1			8

Producers were then asked, ‘Please rate the difficulty of the following aspects of egg production in your cage-free system.’ The aspects which were rated as being more difficult for cage-free compared to cage systems were the prevention of disease (χ^2^ = 19.2, df = 1; *P* < 0.0001), record-keeping (χ^2^ = 11.9, df = 1; *P* < 0.001), achieving good egg production rates (χ^2^ = 16.2, df = 1; *P* < 0.0001), training and knowledge sharing (χ^2^ = 8.6, df = 1; *P* = 0.003), maintaining strict biosecurity (χ^2^ = 6.1, df = 1; *P* = 0.013), litter quality (χ^2^ = 13.9, df = 1; *P* < 0.001) and maintaining good profits (χ^2^ = 31.7, df = 1; *P* < 0.0001). The most difficult of these in cage-free compared with cages were the prevention of disease and maintaining profits; respondents with cage-free systems were 7.9 times more likely to rate maintaining good profits as being more difficult than working in cage systems (OR = 7.9, CI = 3.8–17.0), and 6.2 times more likely to rate the prevention of disease as more difficult than those from cage systems (OR = 6.2, CI = 2.8–16.7). The aspects that were less difficult in cage-free than cage systems were providing environmental enrichment, including perches (χ^2^ = 31.4, df = 1; *P* < 0.0001), nests (χ^2^ = 12.0, df = 1; *P* < 0.001) and pecking objects (χ^2^ = 14.6, df = 1; *P* < 0.001). There was no significant effect of housing system on the perceived difficulty in controlling severe feather-pecking and cannibalism, access to veterinarians, smothering, euthanasia, depopulation and slaughter, or air quality.

Country had a significant effect on the perceived difficulty of controlling severe feather-pecking and cannibalism (χ^2^ = 10.0, df = 4; *P* = 0.04) where Chinese producers were 3.3 times (1.5-7.4) more likely to give a higher difficulty score than producers in Indonesia. Difficulty scores for depopulation and slaughter were affected by country (χ^2^ = 17.5, df = 4; *P* = 0.002). Producers in China (OR = 0.4, CI = 0.2-0.9) and the Philippines (OR = 0.4, CI = 0.1 – 0.97) had lower odds of giving a higher difficulty score than those in Indonesia, however those from Japan had higher odds (OR = 3.4, CI = 1.2 – 9.1).and participants from Indonesia were more likely to rate training and knowledge sharing as more difficult than those from the Philippines (OR = 3.3, CI = 1.1–10). Country had no other effects on the statistical model, suggesting that there were no other differences between countries (however the effect of country could not be tested for all variables due to the small sample sizes). Within countries, the median scores suggest that the cage-free producers in Thailand and Malaysia rated ‘preventing disease’ as the most difficult challenge; cage-free producers in Indonesia had rated it as their second greatest challenge, with ‘accurate record-keeping’ as their biggest challenge. Japan rated ‘training or knowledge sharing’ as their greatest challenges, with ‘maintaining strict farm biosecurity’ as the second greatest challenge in both Japan and Malaysia. These results are presented in Table 3.

**Table 3.** Median scores of the difficulty of practices rated by egg producers (1) Easily achieved, (2) Achievable (3) Unsure (4) Difficult (5) Very difficult

	Indonesia (n = 103/5)		China		Philippines		Thailand		Japan		Malaysia		Total	
			(n = 22/9)		(n = 11/10)		(n = 12/8)		(n = 10/4)		(n = 8/1)		(n = 165/37)	
	Cage	Cage-free	Cage	Cage-free	Cage	Cage-free	Cage	Cage-free	Cage	Cage-free	Cage	Cage-free	Cage	Cage-free
*Preventing disease*	1	3	2	2	2	2	1	3	2	2	2	5	2	2.5
*Litter quality*	2	2	2	2	1	2	1.5	2	2	2	2	2	2	2
*Feather pecking & cannibalism*	2	2	2	2	2	2	2	2	2	2	2	2	2	2
*Maintaining good profits*	2	2	2	3	1.5	2	1.5	3	2	3	2	3	2	3
*Maintaining strict farm biosecurity*	2	2	2	2	2	2	2	2	2	3	2	4	2	2.5
*Air quality*	2	2	2	2	2	1.5	2	2	2	1.5	2	2	2	2
*Enrichment (pecking objects)*	3	2	2	2	3	2	2	1.5	3.5	2	2	1	2.5	2
*Provisions (perches)*	3	2	4	2	5	2	3.5	1.5	4	1	3	1	3.75	1.75
*Provisions (nest boxes)*	2	2	2	1	5	1.5	3.5	2	4.5	1	3.5	1	3.5	1.25
*Accurate record keeping*	1	4	2	2	1	2	1.5	1.5	2	1.5	1.5	1	1.5	1.75
*Access to veterinarians*	2	2	2	2	1	1.5	2	1.5	1	1	2	1	2	1.5
*Preventing smothering*	2	2	2	2	2	2	1	2	2	2	2	1	2	2
*Humane euthanasia*	2	2	2	2	1.5	2	2	2	2	2	2	1	2	2
*Depopulation and/or slaughter*	2	2	2	2	1	2	2	2	3	2.5	2	1	2	2
*Achieving good egg production rates*	2	2	2	3	1	2	1	2	2	2	2	3	2	2.5
*Training or knowledge sharing*	2	2	2	2	1	2	2	2	2	3.5	2	1	2	2

### Solutions to the challenges

The vast majority of responses across all countries identified the top solution as ‘improving on-farm practices’ (comprising 36% of all answers), followed by improved marketing (Table 4). Improving farm practices included efficiencies of operating the farm and employing best practices in bird husbandry and management. Improving farm operations was particularly important in the Philippines, China and Indonesia, while China also focused on improved marketing.

**Table 4.** Cage-free egg producers’ responses to the question ‘What would be some of the solutions to the challenges?’, shown as frequency of theme across all countries

Emerging themes	Philippines	China	Indonesia	Japan	Malay	Thailand	Total
**Information and training for producers** Knowledge/skills transfer (including international), on-farm/operation (health, records, environment, system)	2		1			1	4
**Public education** Animal welfare awareness, reduced criticism (media)		1	1	1			3
**Improved marketing** Product awareness, consumer behaviour, demand, expansion, market differentiation (including high end access)	2	4				3	9
**Egg pricing** Product cost reflective sales price, premium placement	1			1		1	3
**Improving on-farm practices** Improving efficiency, reducing production cost, improving feed conversation ratio, best practice husbandry/management, establish documented procedures, use fences, adequate space and resources, problem solving, egg quality management, sourcing appropriate hen breeds and good quality feed and litter, supplier communications, reduce emissions	5	4	5	3	1	1	19
**Disease prevention and management** Including farm hygiene, establish biosecurity programmes	1	1	2				4
**Increasing investment** Housing, equipment, professional workers, technology	2	1				1	4
**Government support** Financial support (including tax incentives), increased product awareness, establishing animal welfare standards	3	1					4
**Other support** NGO support, financial support, research to improve knowledge	1			1		1	3

### Support needed

In response to the question ‘Do you think farmers need more support to maintain their cage-free farm than is currently available?’, 81.1% (n = 30) of producers responded ‘yes’, 10.8% (n = 4) responded ‘maybe’, and only 8.1% (n = 3) responded ‘no.’ The following questions focused on what type of support was deemed to be needed; ‘improved sales’ was seen as particularly important in the Philippines and Thailand and included topics such as market access, sales certainty, and consumer awareness (Table 5). The Philippines and Indonesia viewed information for producers as important, which included training, flock management, and technical information. There was a focus on improved farm operations in China, which meant the management of the shed, air and litter quality, farm procedures, and disease control. When they were asked who should provide that support, every country except China and Malaysia listed the government as the top stakeholder (Table 6). China viewed the private sector, industry stakeholders and expert organisations and individuals as the top stakeholders that should provide support.

**Table 5.** Egg producers’ perception of the support that is needed to maintain their cage-free systems

Emerging themes	Philippines	China	Indonesia	Japan	Malaysia	Thailand	Total
**Improve sales** Market access, sales certainty, marketing, consumer awareness & behaviour, distribution channels, market policy	6	3	2	1		5	16
**Information for producers** Training, flock management & health education and assistance, technical and financial information	4		3		2	2	11
**Lower farm costs** Consolidate, financial assistance, large-scale production, government support, pricing, low-cost feed, cost-saving	3	2		2		4	11
**Resource availability** Reliable supply of stock, infrastructure, feed	2	1		1		1	5
**Improved farm operations** Air, litter, disease & shed environment management, use of technology, standardising farm procedures		8		1		2	11
**Media** Less criticism, greater accuracy		1		1			2
**Regulation** Government cage-free standards, regulation, trade policy		1	2				3

**Table 6.** Cage-free producers’ responses to the question ‘Who should offer the support that cage-free farmers need?’ by country

Emerging themes	Philippines	China	Indonesia	Japan	Malaysia	Thailand	Total
**Government** Require transition, training, provide capital for transition or new farms, national and regional governments, agricultural departments	10	1	4	4		6	25
**Non-government organisations** Animal activists, NGOs	3	1				1	5
**Private sector** Banks, companies, multinational companies’ global policies, equipment suppliers, retailers, distributors, marketers, hatcheries, integrators, manufacturers	5	3	1		1	3	13
**Industry** Community, poultry veterinarians, other producers, technicians, local villagers	3	3	2	2		1	11
**Experts** Professional organisations, experienced ‘experts’, academics/universities, educational institutions		2	2	1		2	7
**Other** All sectors and associations			2				2

## Discussion

While cage-free housing systems can hold significant animal welfare advantages over cages, there can also be challenges associated with managing these systems. In light of the current trend towards cage-free housing across Asia, this exploratory study aimed to investigate the perspectives of egg producers regarding the main challenges and proposed solutions relating to cage-free systems in China, Indonesia, Thailand, Japan, Malaysia, and the Philippines. To set the scene, questions were asked regarding the main reasons for using cage-free systems in the first place.

### The change to cage-free

The top reasons for producers to use cage-free systems in the present study were current and projected consumer demand. Yang (2020) found that Chinese cage egg producers perceived pressure to move to cage-free egg production from international food companies that had pledged to source cage-free eggs. However, they did not perceive pressure from other stakeholders, including consumers. Several studies, including Alonso *et al.* (2020), have presented evidence for increasing public concern over farm animal welfare globally, including in Asia. In the present study, we did not ask from who the market demand is from; it is possible that the producers were also referring to food companies with cage-free commitments. Nevertheless, public awareness of animal welfare has increased in China (Bayne *et al.* 2015) and there has been increasing public scrutiny of food production practices in China for several years including a focus on food quality, increasing international pressure and the emergence of zoonotic diseases (Littlefair 2006).

### Challenges in cage-free

The animal welfare advantages of cage-free systems compared with cages are generally related to behavioural expression (Hartcher & Jones 2017; Rodenburg *et al.* 2022). This is reflected in the results of the present study, where the provision of enrichment, perches and nests were more challenging to achieve in cage compared to cage-free systems, which is an obvious result since the cage systems in this study were conventional cages that do not have any furnishings. The challenges in cage-free systems that were rated as the most difficult were the prevention of disease, system management, and maintaining good profits, while it was less difficult to provide environmental enrichment in cage-free systems than cage farms. These issues are in line with the known potential difficulties of adopting cage-free systems (European Union 2023), which is also the case for farms in Asia and has been reported as an issue on intensive farms in China (Li 2009).

### Solutions

The number one solution that producers proposed was improving on-farm practices. Rodenburg *et al.* (2022) conducted a similar study to map the solutions to cage-free challenges to encourage a broader uptake of cage-free systems to improve hen welfare. Three groups were formed; the first aimed to identify solutions to current cage-free challenges, the second aimed to identify collaborative opportunities to facilitate the transition to cage-free systems, and the third aimed to identify research needs and the next steps for the ethology community. Participants were ethologists, egg producers and government and non-government organisations. The study found similar results to the present study; one of the main outcomes being that solutions already exist, and dissemination of technical knowledge is needed through education and training of producers. The response themes in that study focused upon technical education and advice for farmers, and collaboration and communication between different stakeholder groups. They also concluded that the way forward includes tackling specific management challenges experienced in cage-free farms, and economic and marketing solutions or support (Rodenburg *et al.* 2022).

A recent study conducted by Shuck-Paim et al. (2021) found that while societal concern for hen welfare is fostering a transition towards cage-free systems globally, a challenge with cage-free housing has been the possibility of higher mortality rates. They conducted a large meta-analysis using data from 6,040 commercial flocks and 176 million hens across 16 countries and provided data on the cumulative mortality at 60 weeks in different housing systems over the years 2000 to 2020. They found decreasing mortality in furnished cages and aviaries over time, but not in conventional cages. The authors explain that the inverse relationship between mortality rates and time is due to experience with the system – meaning that except for conventional cages, mortality rates decrease with increasing experience with the systems over time. Each year of experience was associated with a 0.35–0.65% average drop in cumulative mortality, with no differences in mortality between the cage and cage-free systems in recent years. They also highlight the importance of management knowledge and genetics to accelerate this decline. For example, the reduction in infectious diseases and injurious pecking (two of the biggest risk factors for mortality on cage-free farms), as well as the development of housing design and hen genetics. The results from that study are important evidence that flock management is critical in cage-free systems for hen health and welfare as well as farm productivity (Shuck-Paim et al. 2021).

### Support needed by cage-free producers

The vast majority of farmers felt more support is needed to maintain cage-free farms than currently available, and that the most helpful types of support would be in improving sales, improving farm operations, information for producers, and lower farm costs. Sinclair and Philips (2019) conducted focus groups with leaders in livestock industries in six countries across Asia. Proposed solutions included education, training, and awareness. Another study by the same authors found that senior stakeholders in the Chinese agricultural sector believed they should provide more industry support in the form of training, technical guidance, technological support, animal welfare policies and education. Interest was also shown in the use of successful demonstration farms to lead changes in the industry (Sinclair *et al.* 2022).

Van Dijk *et al.* (2019), ran a large-scale study over 32 months and five European countries. Their study aimed to explore the value of networks in the the layer hen industry in improving the health and welfare of hens, and supported 19 multi-actor networks. It concluded that farmer-led networks can generate effective solutions to animal welfare problems and that greater support should be given to these networks to improve animal welfare strategies and policy development. Van Dijk *et al.* (2019) also recommended that policies be developed to enable access to relatively small amounts of funding for farmer networks seeking to trial innovative activities or procedures, as well as encouraging partnerships between industry, researchers, and technical actors to generate co-operative and innovative partnerships with farmers.

In the present study, all countries except China and Malaysia nominated the government as the most important stakeholder that should be responsible for offering support to cage-free farmers. This could perhaps be because the Chinese government is already highly involved in animal agriculture; increasing agricultural productivity has been a top priority for decades (Li 2009). Sinclair *et al.* (2022) conducted a study in which a questionnaire was given to senior stakeholders in the agriculture sector in China. They found that 88% of participants believed that more support is needed to improve farm animal welfare, but in contrast to the findings in the present study, when they were asked who should provide the support, ‘the government’ was the most frequent response. This may be because participants nominated their own sector as the party responsible for providing support, and the perceived solutions were in the context of what they know and as leaders in that field (Sinclair *et al.* 2022).

### Study limitations

Due to a lack of previous investigations of this nature in these countries, this study serves as an initial exploratory study. Therefore, by nature of design, this study cast a ‘wide net’ to explore a number of topics. As such, it may be used for general information and as a platform from which to conduct further studies on the relevant topics. While there were many benefits to the chosen mixed methodology, it also meant an inability to further question producers in relation to the meaning and details of their questionnaire responses, unlike in a focus group or interview setting. Advised by the exploratory findings in this study, further quantitative investigations may usefully be performed in the region. Additionally, due to the scarcity of cage-free egg farms in the region, there was a small number of producers in the study. This presents a limitation regarding sample size, and basic descriptive statistics are provided rather than statistical testing. Another unavoidable limitation was translating all information twice, from English to the local language and back to English, presenting the possibility of human error.

## Animal welfare implications and conclusion

The adoption of cage-free housing for layer hens can have significant positive impacts on animal welfare. However, there are challenges with cage-free housing that need to be addressed to increase the uptake of these systems and improve animal welfare in Asia. This study provides insights into challenges faced by cage-free egg producers in key Asian countries and proposes solutions to address them. Producers believed that more support is needed, particularly in sales, farm operations and information for producers. These results may aid in providing direction for further studies, and in supplying information to develop relevant initiatives with an emphasis on education and training, to improve animal welfare on cage-free farms and increase the uptake of high welfare cage-free farms across the region.

## Supplementary material

For supplementary material accompanying this paper visit https://doi.org/10.1017/awf.2023.85. click here to view supplementary material
