# Supplementary material for: Improving hen welfare on cage-free egg farms in Asia: Egg producers’ perspectives
Source: Anim Welf. 2023 Sep 22;32:e64. doi: 10.1017/awf.2023.85 (PMC10936346; doi:10.1017/awf.2023.85)
Supplement: Supplementary file 1 [file S0962728623000854sup001.pdf]

Supplementary material

1. Some cage egg farmers are changing to cage-free systems. What do you think are the reasons to use cage-free rather than cage systems? (open ended)
2. What are the main operational challenges in running your cage-free farm? (open ended)
3. Please rate the difficulty of the following aspects of egg production in your cage-free system, where: (1) Easily achieved (2) Achievable (3) Unsure (4) Difficult (5) Very difficult

a. Preventing disease in the birds
b. Maintaining good litter quality
c. Preventing severe feather pecking and cannibalism
d. Maintaining good profits
e. Maintaining strict farm biosecurity
f. Maintaining good air quality
g. Provision of pecking objects
h. Provision of perches
i. Provision of nesting boxes
j. Accurate record keeping
Access to veterinarians
k. Preventing smothering
l. Killing sick or injured hens promptly using humane methods
m. Depopulation and/or slaughter
n. Achieving good egg production rates
o. Training or knowledge sharing with other producers

4. What would be some of the solutions to these challenges (outlined in Q2 + Q3 above)? (open ended)
5. Do you think farmers need more support to maintain their cage-free farm than is currently available? (can select ‘yes’, ‘no’ or ‘maybe’)
6. What support do they need? (open ended)
7. Who should offer that support? (open ended)
8. What support have you received that was helpful? (open ended)
